# Quantum spin Hall insulator in halogenated arsenene films with sizable energy gaps

**DOI:** 10.1038/srep28487

**Published:** 2016-06-24

**Authors:** Dongchao Wang, Li Chen, Changmin Shi, Xiaoli Wang, Guangliang Cui, Pinhua Zhang, Yeqing Chen

**Affiliations:** 1 Institute of Condensed Matter Physics, Linyi University, Shandong 276000, China; 2 School of Physics, Shandong University, Shandong, China

## Abstract

Based on first-principles calculations, the electronic and topological properties of halogenated (F-, Cl-, Br- and I-) arsenene are investigated in detail. It is found that the halogenated arsenene sheets show Dirac type characteristic in the absence of spin-orbital coupling (SOC), whereas energy gap will be induced by SOC with the values ranging from 0.194 eV for F-arsenene to 0.255 eV for I-arsenene. Noticeably, these four newly proposed two-dimensional (2D) systems are verified to be quantum spin Hall (QSH) insulators by calculating the edge states with obvious linear cross inside bulk energy gap. It should be pointed out that the large energy gap in these 2D materials consisted of commonly used element is quite promising for practical applications of QSH insulators at room temperature.

Two-dimensional topological insulators (2D TIs), also called quantum spin Hall (QSH) insulators, have recently attracted quite tremendous attention^1^,^2^. They are usually characterized by the metallic edge states inside bulk energy gap. The gapless edge states are topologically protected by time-reversal symmetry and are robust against nonmagnetic perturbations^3^. They are of promising potential for applications in spintronics and quantum computations, especially, in low-power consumption electronic devices.

The first theoretically predicted and experimentally observed QSH effect is in HgTe/CdTe^4^,^5^ and InAs/GaSb quantum wells^6^,^7^. Nevertheless, their bulk band gaps are so small due to weak spin-orbital coupling (SOC) that the operated temperature is extremely low. Although many film materials have been proposed to be 2D TIs, such as germanene^8^, stanene^9^, bismuthene^10^ and their chemically decorated cases^11^,^12^,^13^,^14^, the experimentally observed QSH effect is just limited in above mentioned two quantum wells. Hence, search and design of 2D TIs with large bulk gap in the commonly used materials is significant for their practical applications at room temperature.

Recently, a new 2D material composed of As element, namely arsenene, is proposed^15^,^16^,^17^, which has a buckled honeycomb configuration like bismuthene. Actually, the arsenene structure corresponds to the bilayer in bulk gray arsenic. Since the interlayer interaction is dominated by the van der Waals force, arsenene can be obtained by exfoliating bulk structure as in the case of phosphorene^18^,^19^. The arsenene has been theoretically predicted to be a normal insulator with trivial band gap, which is quite important for transistors and optoelectronic devices. The topological phase transition from normal to nontrivial phase will be triggered by stretched strain (larger than 11%)^20^. Such large strain, particularly biaxial tensile strain, is very challenging to implement for 2D materials. Thus more useful and feasible methods should be considered to tune the topological phase in arsenene, such as chemical decoration. As previous works reported, surface decoration is an effective way to modulate the structural, electronic and topological properties of 2D systems^21^,^22^,^23^,^24^,^25^.

In this work, the electronic and topological properties of arsenene chemically decorated by halogen atoms are studied via the means of first-principles calculations. We find that the topologically nontrivial phase can be induced in arsenene by surface adsorption of halogen atoms (F, Cl, Br and I). Depending on the important role of SOC, the bulk energy gap is opened relative to the Dirac characteristic without SOC. The values are 0.194 eV, 0.232 eV, 0.240 eV and 0.255 eV for F-, Cl-, Br- and I-arsenene, respectively. Such sizable bulk energy gaps are large enough to realize the QSH effect at room temperature, suggesting that this type material is very potential for future applications in electronic device.

## Computational Methods

First-principles calculations based on density functional theory were performed by the Vienna ab initio simulation package (VASP)^26^. The Perdew-Burke-Ernzerhof (PBE)^27^ generalized gradient approximation (GGA) was used to describe the exchange-correlation potential. The kinetic energy cutoff is 500 eV and the convergence threshold for energy is 10^−6^ eV. The lattice constants and the atom coordinates are fully optimized until the forces on each atom is less than 10^−3^ eV/Å. The Brillouin zone integration is performed with a 17 × 17 × 1 k-mesh for geometry optimization and self-consistent calculations. To simulate isolated thin films, a sufficiently large vacuum space of 20 Å is used to rule out any interactions between the neighboring films. The SOC is included in self-consistent electronic structure calculations. Phonon spectra are calculated for a 5 × 5 × 1 supercell by density functional perturbation theory using VASP and PHONOPY^28^.

## Results and Discussion

**Figure 1:**
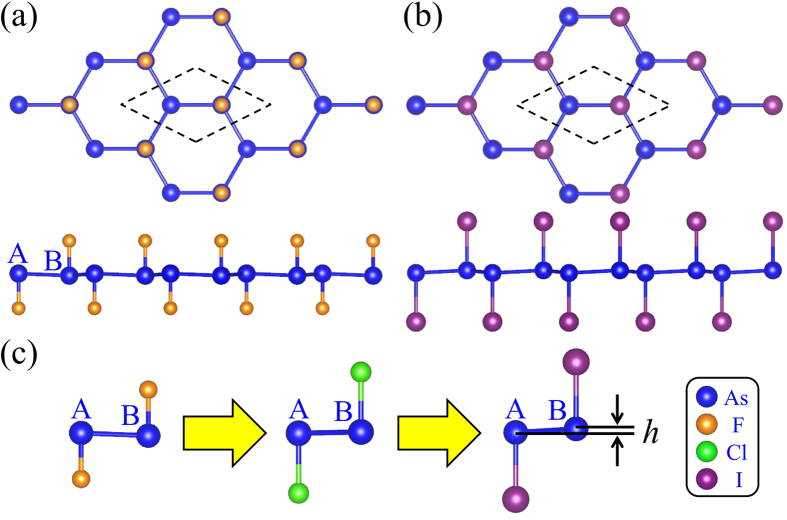
Atomic structures of (**a**) fluorinated arsenene (F-arsenene) film and (**b**) iodinated arsenene (I-arsenene) film. (**c**) Schematic diagram of the evolution of buckling height for halogenated arsenene films.

For the sake of comparison, free-standing arsenene is considered firstly. Based on optimized structure of arsenene, the lattice constant, buckling height and nearest neighbor As-As distance are 3.61 Å, 1.40 Å and 2.51 Å, respectively. Our calculations are in good agreement with the previous theoretical results^15^. Arsenene has a low-buckled structure with *sp*^3^-like hybrid orbital. Due to the reactive surface resulting from dangling bonds, the bare arsenene would be less stable. Therefore, it is highly desirable to stabilize the reactive surface by adsorption of foreign atoms to saturate the dangling bonds. The adsorption of halogen elements has been proposed to be an effective method to modulate structural and electronic properties in various 2D systems^29^,^30^,^31^. In our work, four elements, namely F, Cl, Br and I, are considered. Figure 1(a,b) show the equilibrium structures of fluorinated and iodinated arsenene from top and side views, in which the hexagonal network structure is maintained. Here, the geometry optimizations are performed without and with SOC, respectively. The results show that the effect of SOC is very small on geometry optimization and lattice parameters are almost unchanged. All halogen elements are adsorbed on As atoms with As-X bonds perpendicular to the arsenene sheet. The corresponding structural parameters of F-, Cl-, Br- and I-arsenene are listed in Table 1. The results show that the lattice constants of halogenated arsenene are largely expanded relative to that of bare arsenene. Both lattice constant and As-As bond length increase gradually from F- to I-arsenene. Additionally, As-X bond length increases as the periodic number of halogen elements increases, which results from the increasing atomic radius from F to I atom.

Here, the buckling height *h* is defined as the vertical distance between As_B_ and As_A_ as indicated in Fig. 1(c). As listed in Table 1, the buckling height is 0.108 Å for F-arsenene film, in which the position of As_A_ is higher than that of As_B_, whereas they are 0.050 Å, 0.073 Å, 0.139 Å for Cl-, Br- and I-arsenene, respectively, where As_A_ atom is lower than As_B_ atom. Compared with the case of bare arsenene, the buckling height in halogenated arsenene has been largely compressed. The evolution of buckling height in halogenated arsenene is in relation with the electronegativity of halogens decreasing from 3.98 to 2.66 for F to I element. The similar changing can also be observed in halogenated bismuthene and antimonene film^12^, which is different from that in halogenated group IV elements^32^,^33^. The variation trend of buckling height also suggests that the adsorption of halogen atoms is helpful for structural design in 2D materials.

To evaluate the structural stability of halogenated arsenene, the formation energy *E*_f_ per atom is calculated. The *E*_f_ is defined as *E*_f_ = (*E*_X-As_ − *E*_As_ − 2*E*_X_)/2, where *E*_X-As_ is the energy of halogenated arsenene, *E*_As_ is the energy of pure arsenene, and *E*_X_ is the binding energy per atom of an X_2_ molecule. All the calculated *E*_f_ values listed in Table 1 are distinctly negative, indicating exothermic adsorption and more stable structure with chemical bonding between halogen and As atoms. Moreover, note that the π bonds in arsenene originate from the overlapping of 4*p*_*z*_ orbitals of the As atoms. The *p* orbitals perpendicular to the plane of six-atom ring combine to form a weak and extensive π-bonding network. However, when all of the As atoms are halogenated, strong *σ* bonds form between As and F (Cl, Br and I) atoms and the π-bonding network is broken. Saturating the dangling bonds by surface decoration is helpful to enhance the immunity of arsenene to ambient conditions.

**Figure 2:**
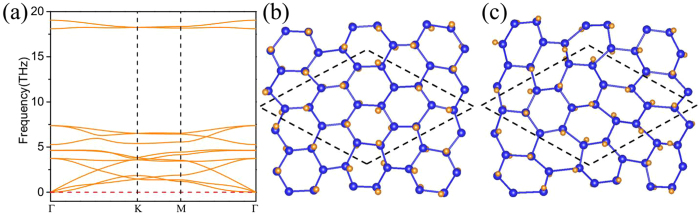
(**a**) Phonon spectrum for F-arsenene film. Corresponding MD simulation of the structure for F-arsenene film (**b**) at 300 K and (**c**) at 400 K. The dashed line indicates a supercell with a 3 × 3 × 1 unit cell.

In addition, the kinetic stability of these halogenated arsenene films is further confirmed by calculating the phonon spectra. For F-arsenene film as shown in Fig. 2(a), there is no imaginary frequency along all momenta, which indicates that this structure is kinetically stable. For Cl-arsenene film as shown in Fig. S1(a), there is small imaginary frequency near the Γ point. In fact, this small imaginary frequency is a common issue in first-principles calculation for 2D materials, which is sensitive to the details of the calculation and in some cases will disappear, so Cl-arsenene film is also dynamically stable. However, for Br- and I-arsenene films, more imaginary frequency appears at the K point as shown in Fig. S1(b,c), indicating dynamically unstable structures. To verify the thermal stability of these films, we also performed *ab initio* molecular dynamics (MD) simulations using a supercell of 5 × 5 × 1 unit cells at various temperatures. Taking F-arsenene film for example, we find in Fig. 2(b) that the hexagonal lattice geometry is distorted slightly and the movement of F atoms is small at 300 K, indicating F-arsenene film is thermally stable at room temperature. However, when the temperature is increased to 400 K, large deformation occurs with the inversion symmetry destroyed as shown in Fig. 2(c). Other halogenated arsenene films are also found to be stable at room temperature, whereas large distortion appears at higher temperature. Our results are similar to those of previous report for H-adsorbed Bi film^12^.

**Figure 3:**
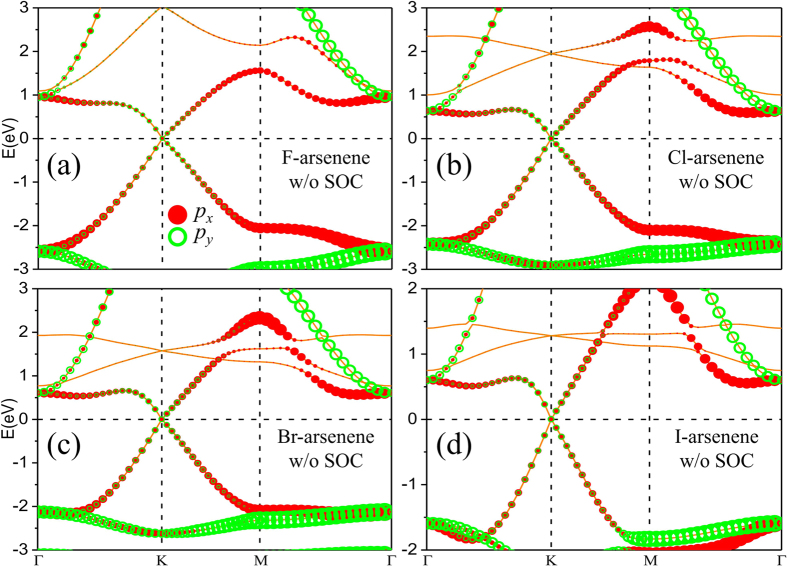
Electronic band structures with orbital projections for (**a**) F-arsenene, (**b**) Cl-arsenene, (**c**) Br-arsenene and (**d**) I-arsenene films without SOC. The radius of red dot and blue circle represents the weight of *p*_*x*_ and *p*_*y*_.

To reveal the influence of surface decoration on the electronic properties of arsenene, the electronic band structure calculations are performed. For the purpose of comparison, the band structures of pure arsenene are also calculated. The results show that pure arsenene is normal insulator with indirect band gap of 1.61 eV and 1.48 eV for the case without and with SOC, respectively. Next turn our attention upon the electronic properties of halogenated arsenene of which the band structures without SOC are presented in Fig. 3. In contrast to that large band gap would be obtained for graphene and silicene by chemisorption of halogen atoms, Dirac point type characteristic is formed with linear cross at the Fermi level at the K point in the Brillouin zone of F-, Cl-, Br- and I-arsenene. Compared with pure arsenene, halogenated arsenene becomes Dirac materials in the absence of SOC, which suggests that the electronic properties could be strongly modulated by chemisorption. To analyze deeply the orbital contribution to energy bands, the orbital-resolved band structures for halogenated arsenene, namely projecting various orbitals on each band, can also be observed in Fig. 3. Different from that the bands of pure arsenene around the Fermi level are mostly contributed by *p*_*x*_, *p*_*y*_ and *p*_*z*_ orbitals of As atoms, in halogenated arsenene the bands near Fermi level are mainly composed of *p*_*x*_ and *p*_*y*_ orbitals of As atoms with the *p*_*z*_ orbital removed due to the saturation of dangling bonds.

**Figure 4:**
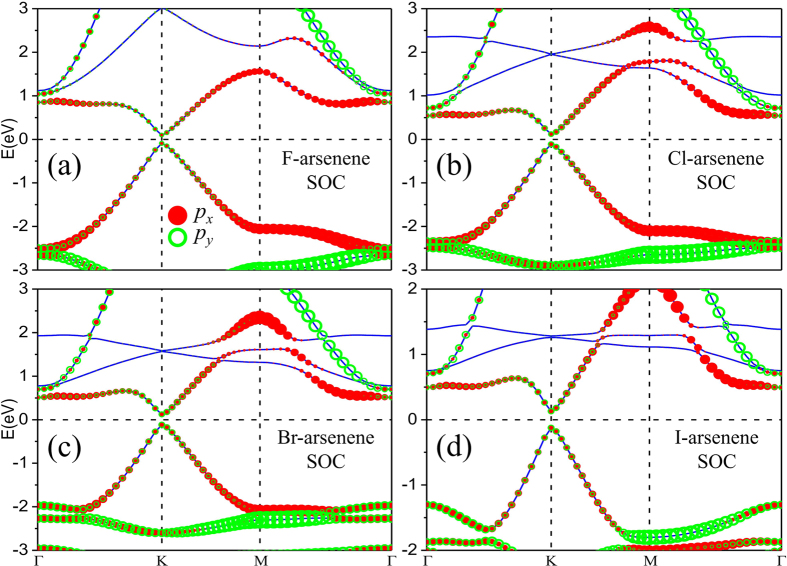
Electronic band structures with orbital projections for (**a**) F-arsenene, (**b**) Cl-arsenene, (**c**) Br-arsenene and (**d**) I-arsenene films with SOC. The radius of red dot and blue circle represents the weight of *p*_*x*_ and *p*_*y*_.

As we know, in QSH insulators the SOC plays an indispensable role. On the one hand, the strong strength of SOC in heavy elements could induce band inversion to result in QSH phase^34^,^35^. On the other hand, the SOC would induce energy gap in gapless 2D systems^36^,^37^. When the SOC is switched on in our calculations, the band structures of F-, Cl-, Br- and I-arsenene are shown in Fig. 4. One can obviously see that those degenerated bands around Fermi level are lifted out and split into two single states. Consequentially, including SOC opens up the energy gap at the K point for halogenated arsenene with different values varied from 0.194 eV to 0.255 eV as listed in Table 1. The enhancement of energy gap from F- to I-arsenene is in connection with the increasing SOC strength from F to I atom. Then the orbital projections on each band with SOC are also plotted for F-, Cl-, Br- and I-arsenene as shown in Fig. 4. Compared with the case without SOC, the orbital contribution to band does not be apparently changed. For example, the bands near the Fermi level mainly originate from *p*_*x*_ and *p*_*y*_ orbitals for F-arsenene in both Figs 3(a) and 4(a). The role of SOC in halogenated arsenene is to open up energy gap, which is similar to silicene and stanene that are 2D TIs^8^,^9^.

To distinguish nontrivial insulators from ordinary insulators, the Z_2_ topological invariant is calculated^38^. The value of 0 characterizes an ordinary insulator, while the value of 1 indicates a nontrivial phase. For the 2D TI phase, the topological invariant is calculated from the parities of the Bloch wave functions for occupied bands at time-reversal invariant momenta (TRIM) points, one Γ and three M points, as


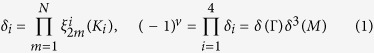


where 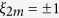 denotes parity eigenvalues and *N* is the number of the occupied bands. The calculated Z_2_ number (*v*) for halogenated arsenene films is listed in Table 1. The Z_2_ values from F- to I-arsenene are nonzero indicating nontrivial insulators.

**Figure 5:**
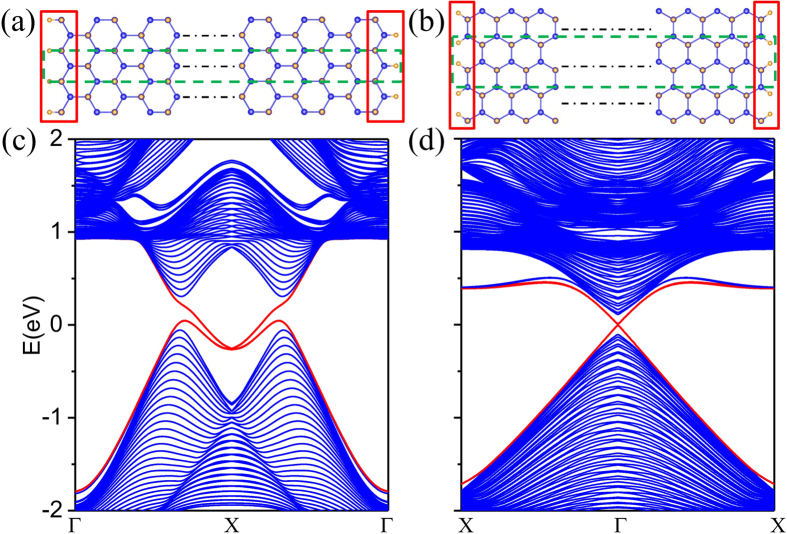
Atomic structures of ribbons with (**a**) zigzag and (**b**) armchair edges for F-arsenene. The green dotted lines represent the unit cell of ribbons, and the red solid lines indicate the atoms on different edges. Corresponding band structures of ribbons with (**c**) zigzag and (**d**) armchair edges with X = π/L where L is the width of nanoribbon.

Compared with conventional insulator, the 2D TIs possess an outstanding feature, namely, topological protected conducting edge states on the boundary. To see these topological features explicitly, we perform calculations of the edge states by cutting 2D films into nanoribbon. Both zigzag and armchair shaped edges are considered where all the edge atoms are passivated by halogen atoms to eliminate the dangling bonds. The widths of the ribbons are taken to be fairly large to avoid the interaction between the two edges. The nanoribbon models of F-arsenene with zigzag and armchair edges where the edge atoms are saturated by F atoms are shown in Fig. 5(a,b), respectively. The corresponding band structures are illustrated in Fig. 5(c,d), in which we can clearly observe the topological edge states (red lines) for both zigzag and armchair shaped nanoribbons, further confirming nontrivial phase of F-arsenene. Such edge states form Kramers pairs with spin currents flowing oppositely for opposite directions of spins, which are important for the applications in electronic devices^39^, due to their robustness against back-scattering. Moreover, the calculations of edge states for Cl-, Br- and I- arsenene are also performed. From the band structures of the nanoribbons which are very similar to those of F-arsenene, the edge states connecting the bulk conduction and valence bands are obviously seen to linearly cross at the X and Γ points for zigzag and armchair shaped nanoribbons, further confirming nontrivial phase of Cl-, Br- and I- arsenene.

Here we would like to emphasize that there is one important difference between the edge states of the QSH phase and the graphene. The edge states in the QSH phase carry helical spin currents, and circulate along the whole edge around, in despite of the details (e.g. the shape) of the edge. In graphene the existence of edge states crucially depends on the edge shape, where the zigzag edge has edge states while the armchair edge does not^40^. Furthermore, it should be pointed out that different from the original Kane-Mele model which is a 2-band model for single orbital (*p*_*z*_ of C atom in graphene) nearest-neighbor hopping on a hexagonal lattice, our proposed systems actually have 4 bands with *p*_*x*_ and *p*_*y*_ orbitals, similar to chemically modified Bi or Sb cases or Bi, Sb and other elements on Si substrates where the *p*_*z*_ orbital is filed out by “orbital filtering effect”^41^. Up to date, most of TIs that have been predicted with sizable bulk energy gap are composed of heavy elements, such as Sn, Pb, Sb and Bi and so on, because of large SOC strength. Although some 2D film materials, such as chemical decorated silicene and germanene, have been proposed to realize QSH effect, the bulk energy gaps in these systems are too small to stabilize the edge current against nonmagnetic disorder. The bulk energy gap is highly enlarged in arsenene modified by halogens. Such gap is much larger than the thermal motion energy (k_B_T, ∼26 meV) at room temperature, which is significantly potential for TI-based electronic devices at room temperature.

In privious works, it has been revealed that the mechanism of nontrivial topology in some pristine and functionalized 2D materials originates from the *s*-*p*_*xy*_ type band inversion at the Γ point^9^,^42^,^43^,^44^,^45^, which is similar to that in a HgTe quantum well^46^. However, the origin of nontrivial topology in halogenated arsenene results from the massive Dirac point and there is no band inversion, where the mechanism is similar to that in functionlized Bi/Sb films^12^,^13^,^14^,^47^. Around Fermi level, there are massive Dirac cones at the K point and nearly flat bands (the second band below the Fermi level) in the band structures of halogenated arsenene. Massive Dirac cones and flat bands are mainly contributed by *p*_*x*_ and *p*_*y*_ of As and halogen atoms in the band components, which is distinguished from those in graphene and silicene mainly composed of *p*_*z*_ orbital of C and Si atoms.

## Conclusions

Using density functional theory (DFT) computations, we investigate the electronic and topological properties of halogenated arsenene. New TI materials with sizable band gaps are predicted in F-, Cl-, Br- and I- arsenene, in which the role of SOC is to open up bulk energy gap. The energy gaps vary from 0.194 eV to 0.255 eV for halogenated arsenene. The calculations of helical edge states of nanoribbons confirm the existence of helical edge states with conducting channels. Our results show that halogenation is one of effective methods to modulate the quantum phase from normal to nontrivial insulator. Halogenated arsenene is strongly suggested to be an ideal host for the QSH effect, thus providing a pathway to the spintronics and quantum computations.

## Additional Information

**How to cite this article**: Wang, D. *et al*. Quantum spin Hall insulator in halogenated arsenene films with sizable energy gaps. *Sci. Rep*. **6**, 28487; doi: 10.1038/srep28487 (2016).

## Supplementary Material

Supplementary Information

## Figures and Tables

**Table 1:** Lattice parameters for halogenated arsenene.

Structure	*a*(Å)	*d*_As-As_(Å)	*d*_As-X_(Å)	*h*(Å)	*E*_f_(eV)	*E*_*g*_(eV)	Z_2_
F-arsenene	4.57	2.64	1.81	0.108	−2.364	0.194	1
Cl-arsenene	4.63	2.67	2.22	0.050	−0.783	0.232	1
Br-arsenene	4.64	2.68	2.38	0.073	−0.568	0.240	1
I-arsenene	4.68	2.70	2.59	0.139	−0.265	0.255	1

The *a*, *d*_As-As_, *d*_As-X_, *h*, *E*_f_, *E*_g_ and Z_2_ stand for the lattice constant, As-As bond length, As-X bond length (X = F, Cl, Br and I), buckling height, formation energy, SOC-induced energy gap and topological invariant.
