# Supplementary material for: Quantum spin Hall insulator in halogenated arsenene films with sizable energy gaps
Source: Sci Rep. 2016 Jun 24;6:28487. doi: 10.1038/srep28487 (PMC4919688; doi:10.1038/srep28487)
Supplement: Supplementary Information [file srep28487-s1.doc]

**Supporting Information**

Quantum spin Hall insulator in halogenated arsenene films with sizable energy gaps

Dongchao Wang, Li Chen*, Changmin Shi, Xiaoli Wang, Guangliang Cui, Pinhua Zhang and Yeqing Chen

Here we provide the phonon dispersions for other three halogenated arsenene films except fluorinated arsenene.

Phonon spectra are calculated for a 5×5×1 supercell by density functional perturbation theory using VASP and PHONOPY.


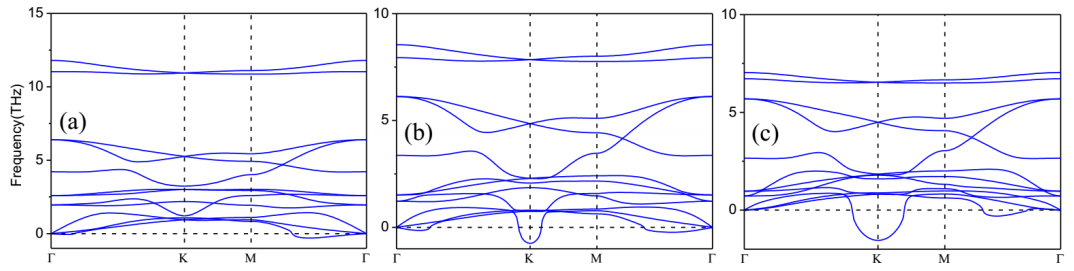


Figure S1. The phonon dispersions of (a) Cl-arsenene, (b) Br-arsenene and (c) I-arsenene.
